# Genetic analyses in Lake Malawi cichlids identify new roles for Fgf signaling in scale shape variation

**DOI:** 10.1038/s42003-018-0060-4

**Published:** 2018-05-31

**Authors:** R. Craig Albertson, Kenta C. Kawasaki, Emily R. Tetrault, Kara E. Powder

**Affiliations:** 1 Department of Biology, University of Massachusetts, 611 North Pleasant Street, Amherst, MA 01003 USA; 2 Graduate Program in Molecular and Cellular Biology, University of Massachusetts, 611 North Pleasant Street, Amherst, MA 01003 USA; 3 Department of Biological Sciences, Clemson University, 190 Collings Street, Clemson, SC 29634 USA

## Abstract

Elasmoid scales are the most common epithelial appendage among vertebrates, however an understanding of the genetic mechanisms that underlie variation in scale shape is lacking. Using an F_2_ mapping cross between morphologically distinct cichlid species, we identified >40 QTL for scale shape at different body positions. We show that while certain regions of the genome regulate variation in multiple scales, most are specific to scales at distinct positions. This suggests a degree of regional modularity in scale development. We also identified a single QTL for variation in scale shape disparity across the body. Finally, we screened a QTL hotspot for candidate loci, and identified the Fgf receptor *fgfr1b* as a prime target. Quantitative rtPCR and small molecule manipulation support a role for Fgf signaling in shaping cichlid scales. While Fgfs have previously been implicated in scale loss, these data reveal new roles for the pathway in scale shape variation.

## Abstract (web summary)

Craig Albertson et al. report a study of the genetic basis of scale shape variation in cichlids from Lake Malawi. They find 40 QTL for scale development, most of which are specific to distinct body locations, and a role for Fgf signaling in shaping cichlid scales.

## Introduction

An expansion of the integument represents a key innovation of vertebrates that has contributed to their evolutionary success^1,2^. Compared to invertebrate chordates, vertebrate skin is both thicker and more complex. The dermis is particularly rich in different cell types and structures, including fibroblasts, mast cells, macrophages, pigment cells, and scleroblasts, as well as blood and lymphatic vesicles and nerves. The epidermis is also stratified into structurally and functionally distinct layers, with mitotic cells constituting deeper layers and keratinized cells forming a superficial protective layer (review by refs. ^3,4^). Beyond this tissue-level diversity, myriad organs may arise from reciprocal interactions between the epidermis and dermis. Referred to as integumentary or epithelial appendages, these structures include scales, teeth, feathers, horns, nails, claws, beaks, and glands. They are often specific to different vertebrate lineages, and collectively help to define vertebrate disparity. Notably, all epithelial appendages share a common developmental origin and begin as a localized thickening of the epidermis during the placode stage. The type of epithelial appendage to form depends largely on the specific signaling molecules and transcription factors that are expressed in the overlaying epidermis and underlying dermis.

By far the most common epithelial appendage in vertebrates are scales. Scales are also the most ancestral epithelial appendage, and share deep homology with more derived types^5–7^. In contrast to amniote epithelial appendages, very little is known about the molecular basis of scale morphogenesis in fishes^8,9^. Even less is known about the mechanisms that underlie variation in scale shape, despite the tremendous diversity in scale shape among fishes. While shape differences between major scale types are conspicuous (e.g., tooth-shaped placoid scales versus cycloid-shaped elasmoid scales), variation also exists within scale types, including disparity in size (e.g., large scales in tarpon, tiny scales in tuna^10^), shape (e.g., among mullet species^11^), and the presence/absence of scales (e.g., loss in catfish^12^). Notably, scales can also exhibit considerable variation across the body within an individual. For example, flatfish possess different scale types on their blind versus eyed sides^13^, tuna tend to possess large scales on their bodies at the region of maximum girth to reduce drag^10^, and bluegill sunfish exhibit subtle but measurable differences in scale shape across the body^14^. Given the impressive diversity in scale shape among (and within) fishes, it is surprising that the molecular basis for scale shape evolution is virtually unknown.

Much of what we know about the evolution of scales is limited to scale loss. For example, genome-wide sequence data implicated loss of secretory calcium-binding phosphoprotein (SCPP) genes in the evolution of scale loss in catfish^12^. In addition, loss-of-function mutations in Fgf receptors were found to be associated with independent scale loss in two cyprinid lineages (domesticated carp^15^; *Phoxinellus*^16^). These insights are consistent with roles for Fgf signaling in the development of other epithelial appendages, including feathers^17^ and turtle shell scutes^18^. In instances of scale loss, the causative mutation likely disrupts placode formation, leading to loss of the organ. While such insights contribute to a deeper understanding of early scale development/patterning, they are less informative with respect to differential growth/mineralization that leads to variation in scale shape. Here we seek to expand our understanding of scale evolution by examining the genetic basis of scale shape among Lake Malawi cichlids, a model system for extensive and rapid evolution of myriad mineralized (e.g., craniofacial^19–21^), serially homologous (e.g., paired fins^22,23^), and epithelial appendage traits (e.g., teeth and tastebuds^24,25^). We show that closely related species exhibit measureable differences in scale shape, and that scale shape variation between and within individuals has a tractable genetic basis. Results from fine mapping, quantitative rtPCR, and small molecule manipulation suggest that Fgf signaling plays an important role in determining scale shape. Taken together, these data offer novel insights into the development and evolution of fish scales.

## Results

### Scale shape variation

**Fig. 1:**
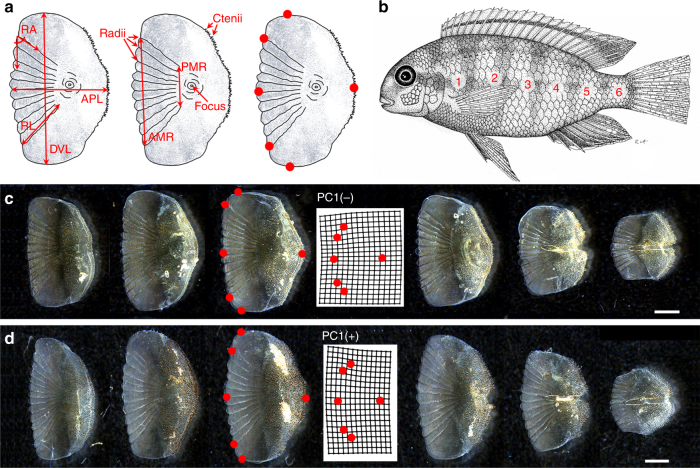
Scale shape variation for two cichlid species. **a** Measures for scale shape, including landmarks for geometric morphometrics. **b** Position along the anterior–posterior axis where scales where taken for imaging and measurements. **c** Scales 1–6, left to right, for *Labeotropheus fuelleborni* **d** Scales 1–6, left to right, for *Tropheops* red cheek. Mean scale shapes for *L*. *fuelleborni* (*n* = 12) and *T*. red cheek (*n* = 12) along PC1 are shown in the center of **c** and **d**, respectively. Scale bars = 1 mm

We focused on two species of Lake Malawi cichlids, *Labeotropheus fuelleborni* and *Tropheops* red cheek, which represent closely related genera and near ecological competitors. Both species occupy the shallow, near-shore, rocky habitat, and as adults forage mainly upon filamentous alga that is detached from rocks. However, the specific mode of food collection is different between the two species. Whereas *L. fuelleborni* possess wide mouths and crop algae while swimming parallel to the substrate, *T*. red cheek have small, narrow mouths and nip strands of algae with a twist and lateral jerking motion^26,27^. Consistent with these differences in feeding mode, *L. fuelleborni* and *T*. red cheek exhibit measurable differences in several phenotypic traits^23,28–32^. Upon preliminary inspection, they also appeared to exhibit general differences in scale shape (Fig. 1). We sought to more formally describe and quantify these differences.

One angle and seven linear measurements were made on six different scales positioned along the flank of each fish (Fig. 1). Parental species exhibited significant differences in each measure for at least one scale (Supplementary Figs. 1–10, *n* = 12 for each species). The most prominent differences were noted for the total height of the scale (dorsal-ventral length), the length of the anterior margin of the radii, and the number of radii (see statistical significance in Supplementary Figs. 1–8). These measures were particularly distinct for scales 3 and 5. For each of the above measures, *T*. red cheek scales consistently had higher values than *L. fuelleborni* scales. Moreover, for nearly all linear measures of scale shape, F_2_ animals (*n* = 256) exhibited intermediate trait values (Supplementary Figs. 1–8), which is consistent with an additive mode of inheritance.

Shape was also assessed in the parental species and the F_2_ population via geometric morphometrics. Parental species differed significantly in their position along the first principal component axis, which explained 38.2–50.9% of the total variation depending on the scale position analyzed (Table 1). This axis mainly described variation in scale height, as well as how close the radii extend to the dorsal and ventral margins of the scales (Table 1; Fig. 1c; Supplementary Fig. 11). As with the linear measures, scales 3 and 5 were among the most divergent in shape space (Table 1).

**Table 1:** Quantification of scale shape variation via landmark-based geometric morphometrics

Scale	PC1	PC2	PC3	PC1-PC3
P1	38.2%, 0.0623^a^	23.4%, 0.141^c^	17.0%, 0.152^b^	*F*_1,21_ = 3.426, *p* = 0.0381
P2	45.1%, 0.0237^a^	28.6%, 0.0886^b^	11.4%, 0.974^c^	*F*_1,21_ = 3.448, *p* = 0.0374
P3	50.2%, 6.80e-06^a^	25.3%, 0.871^b^	14.6%, 0.102^c^	*F*_1,21_ = 19.05, *p* = 5.95e-06
P4	45.1%, 0.00288^a^	22.7%, 0.281^b^	16.3%, 0.169^c^	*F*_1,21_ = 6.20, *p* = 0.00407
P5	50.6%, 0.00380^a^	16.8%, 0.742^b^	15.5%, 0.879^c^	*F*_1,21_ = 3.285, *p* = 0.0433
P6	50.9%, 0.838^a^	24.6%, 0.794^c^	13.3%, 0.125^b^	*F*_1,21_ = 0.816, *p* = 0.501
P1-6 combo	68.9%^a^	11.9%^b^	7.10%^c^	
F_2_3	37.4%^a^	30.4%^b^	12.1%^c^	
F_2_5	46.0%^a^	21.9%^b^	13.1%^c^	
F_2_1-6 combo	70.9%^a^	13.1%^b^	7.10%^d^	

Results are reported for both parental (e.g., P1–6) and F_2_ (e.g., F_2_3 and 5) scales. For parental scale, percent variance explained (PVE) and *p*-value from *t*-test are reported for PC1, PC2, and PC3 comparing shapes of *L. fuelleborni* and *T*. red cheek. MANOVA (Wilks test) results are also reported for each scale. PVE is reported when all scales were analyzed together, and for F_2_ scales 3 and 5, which were used for QTL analyses

^a^ Describe variation in scale height at the anterior margin of the radii. Note that in every analysis this is PC1. Negative values correspond to shorter scales, whereas positive values correspond to a taller scale

^b^ Describe variation in scale length, as well as in height at the anterior radii margin. This type of variation is mainly captured on PC2. Negative values correspond to wider and taller scales, whereas positive values correspond to a shorter and narrower scale

^c^ Describe asymmetry in the midline of the scale relative to scale height at the anterior margin of the radii. Negative values correspond to scales where more radii are dorsal to the midline, whereas positive values indicate scales where more radii are ventral to the midline. This type of variation is mainly captured on PC3

^d^ Describes variation in curvature on anterior edge of radii. Negative values describe curvature that is convex, whereas positive values indicate a relatively flat anterior edge

Results are reported for the first three PC axes in the F_2_ population, which collectively explain ~80% (Table 1; Supplementary Figure 11) of the total shape variation. Because they were consistently the most divergent between parental species in linear and geometric measures, we focused on scales 3 and 5 for these analyses. Variation along each axis was largely consistent between scales. PC1 in both scales explained variation in height at the anterior margin of the radii. PC2 described variation in scale length, as well as in height at the anterior radii margin. PC3 described an asymmetry in the midline of the scale relative to scale height at the anterior margin of the radii (i.e., negative values correspond to scales where more radii are dorsal to the midline, whereas positive values indicate scales where more radii are ventral to the midline). Scores along these PC axes were used for subsequent QTL analyses.

Finally, we assessed disparity in scale shape within individual fish. Scales represent serially homologous structures that have become individualized in many species such that scales on one body region look different from those on another. This difference is most conspicuous in terms of scale size (i.e., smaller anteriorly to facilitate streamlining), but we hypothesized that there might also exist a difference in shape. Using the same set of landmarks as above, we assessed shape variation across all six scales and compared levels of disparity between individuals. Notably, *T*. red cheek exhibited higher levels of disparity on average (ProcVar = 0.00855), compared to *L. fuelleborni* (ProcVar = 0.0061; paired *t*-test, *p* = 0.029). Disparity was also calculated for F_2_ hybrid animals, and used for subsequent QTL mapping to see if we could detect a genetic component for this trait.

### QTL mapping of scale shape variation

**Fig. 2:**
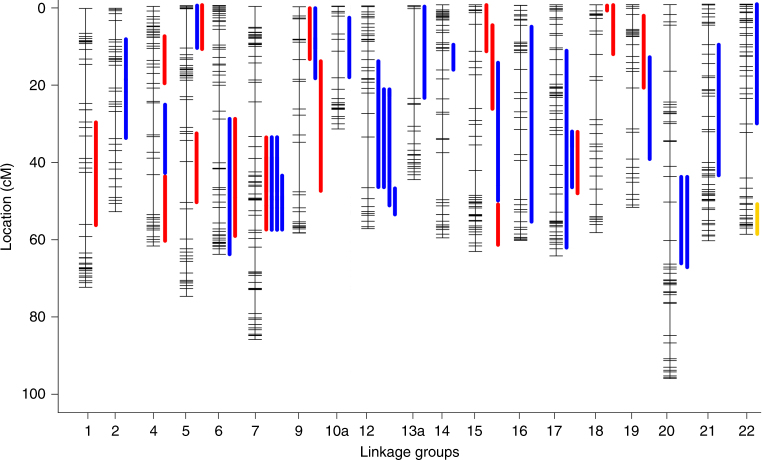
QTL map for scale shape variation. Results are shown for various measures of scale 3 (red, *n* = 16), scale 5 (blue, *n* = 25), and scale shape disparity across the body (yellow, *n* = 1). Bar lengths correspond to 95% confidence intervals. Full data are provided in Supplementary Table 1

QTL analysis revealed numerous regions of the cichlid genome that underlie divergence of specific phenotypic traits (Supplementary Table 1; Fig. 2). In total, 42 significant QTL (38 at the <0.05 genome-wide level, 4 at the < 0.10 genome-wide level) were identified representing all traits measured—16 QTL for scale 3, 25 QTL for scale 5, and one QTL for scale shape disparity. QTL intervals for scale 3 and 5 only overlapped at seven loci (<44% for scale 3, <30% for scale 5), suggesting a high degree of genetic modularity for scale shape across the body. In fact, no trait shared a common set of QTL for both scales. Extreme instances of modularity include traits for which several QTL were detected for one scale but none were detected for the other (e.g., radii length, Supplementary Table 1), and traits where several QTL were detected for each scale but none overlapped (e.g., radii angle, Supplementary Table 1). Further, QTL were only detected for five traits for scale 3, whereas QTL for eleven traits were detected for scale 5. There was also considerable variation in the number of QTL detected for different traits. Whereas only a single QTL was detected for the number of radii (on scale 5), seven QTL were detected for depth of the scale at the anterior margin of the radii (four for scale 3 and three for scale 5). Thus, certain scales and traits appear to have more tractable genetic bases than others.

Significant LOD scores were found on 19 of the 25 linkage groups (Supplementary Table 1; Fig. 2). Several linkage groups (LGs) possessed overlapping QTL. For example, four QTL overlapped on LG 7, including traits for both scales 3 and 5. LGs 12 and 17 each had three overlapping QTL, and LGs 5, 6, 9, 15, 18, 19, and 20 all had two overlapping QTL. For many of these intervals (e.g., LGs 5, 6, and 20) QTL confidence intervals were nearly identical, which suggests that the associated traits may share a common genetic basis—e.g., pleiotropy.

All significant QTL were of small to modest effect, explaining between 6–13% of the variation in the F_2_ animals. Modes of inheritance ranged from additive to dominant to overdominance. For many traits the allele effects were not consistent across QTL. However, the *T*. red cheek allele generally increased trait values for phenotypes associated with scale height. This trend held for 9 of 11 QTL, including those for overall height, height at the anterior margin of the radii, and PC1, which largely explained variation in scale height at the anterior margin of the radii. These data are consistent with *T*. red cheek exhibiting deeper scales than *L. fuelleborni*, and the consistent direction of allele effects suggests that divergent selection might be acting on this dimension of scale shape^19,33^. Finally, the *T*. red cheek allele was found to increase scale shape disparity and to be dominant to the *L. fuelleborni* allele. This pattern is also consistent with the observation that *T*. red cheek exhibited greater disparity in scale shape than *L. fuelleborni*. In all, these data suggest that scale shape variation between closely related cichlid species has a relatively complex genetic basis, however for certain traits such as scale height trends point to divergent selection acting on scale shape variation.

### fgfr1b as a candidate for scale shape QTL on LG7

A QTL hotspot was detected on LG7, where four QTL overlapped between 43.7–57.9 cM. Each of these QTL influenced traits that affect the overall dimensionality of the scale, and include height of scale 5, length of scale 5, and PC2 for scales 3 and 5. As noted above, PC2 describes variation in scale length, as well as in height at the anterior margin of the radii (Supplementary Figure 11). Thus, this locus appears to be important for mediating broad and general aspects of scale shape. Moreover, the *T*. red cheek allele was consistently associated with increased trait values for each QTL.

This overlapping QTL interval corresponds to four physical sequence scaffolds and ~20 Mb in the Lake Malawi genome. To narrow this large interval to a smaller set of candidate loci, we utilized genome scans from natural populations of *L. fuelleborni* and *T*. red cheek to search for loci with outlier F_ST_ values (>0.6, after ref. ^34^) on these scaffolds (data published in ref. ^35^). Ninety-four such SNPs associated with 68 protein-coding genes were detected. Table 2 presents a reduced set wherein only the SNP with the highest F_ST_ value is shown per gene. Several candidate genes with outlier F_ST_ values were noted in this region, including *tbx3a, tbx5a, bmp1a, col1a1-like*, and *alx1* (boldfaced in Table 2). Each of these factors have been implicated in bone, skin, and/or placode development, and it is possible that the causative mutation(s) for scale shape variation is associated with one or more of these genes. However, two loci stood out as particularity promising candidates. First, on scaffold 0, three SNPs with high F_ST_ values were located just 5ʹ and 3ʹ of *ephrin A5a* (Table 2). This gene encodes a protein that belongs to the ephrin-A subclass of ephrin ligands that can bind to both EphA and EphB2 receptors^36^. Ephrins play myriad roles in development, and have been shown specifically to regulate placode development and polarity in another epithelial appendage, feathers^37,38^. Second, on scaffold 45, three SNPs with outlier F_ST_ values were located just 5ʹ to the first of three *fgfr1b* paralogs tandemly arrayed along the scaffold (Table 2). This region is an especially attractive candidate as *fgfr1a* has been shown to (1) be expressed in developing zebrafish scales, (2) underlie scale development in zebrafish, and (3) underlie the evolution of scale loss in two different lineages of fish^15,16^. Finally, *ephrin A5a* and *fgfr1b* consistently define the LOD peak for each QTL in this region, which plateaus between 45–50 cM (*ephrin A5a* is at 45 cM and *fgfr1b* is at 50 cM), whereas other candidate genes consistently fall outside this LOD peak (Table 2).

**Table 2:** Fine mapping and candidate gene identification in the QTL hotspot on LG7

Physical scaffold and nucleotide position for genetic markers are reported, as well as the F_ST_ values from genome scans comparing wild caught *L. fuelleborni* and *T*. red cheek. The location of the SNP relative to the closest gene is also presented (e.g. 5ʹ/3ʹ of the gene or within an intron). A heat map is presented here for scale 3 PC2 LOD scores, which peak between 45–50 cM. Candidate genes within the 95% confidence interval are boldfaced and colored according to LOD scores. Both *efnA5a* and *fgfr1b* stand out as prime candidates through this approach

**Fig. 3:**
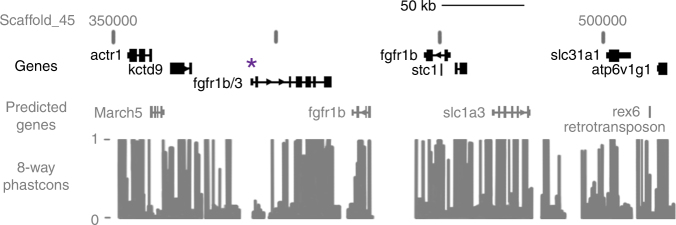
Schematic of *fgfr1b* candidate interval. Genes annotated in the *Maylandia zebra* genome are shown in black, and based on synteny with zebrafish. Genes predicted through BLAST are shown in gray. Phastcons scores indicate sequence conservation among 5 cichlid genomes (*M. zebra, Pundamilia nyererei, Astatotilapia burtoni, Neolamprologus brichardi*, and *Oreochromis niloticus*), medaka, stickleback, and zebrafish. Phastcons scores of “1” are complete conserved, while nucleotides with a “0” are not conserved across the eight species. Asterisk indicates the paralog for which primers were designed for qPCR

We chose to focus subsequent analyses on *fgfr1b* for a few reasons. First, this pathway has been implicated in the development and evolution of placodes in general^39,40^, and of elasmoid scales in particular^15^. Second, this interval constitutes a relatively large target for selection with at least two and possibly three tandem duplicates of *fgfr1b* on scaffold 45 between 391880–452990 bp (Fig. 3), which appears to be conserved across East African cichlids. Recent genomic analyses have provided compelling evidence for selection acting on ancestrally duplicated gene families in Lake Malawi cichlids^41^.

### Epistasis between fgfr1b and fgf20a

Our knowledge of scale development has benefited from developmental and genetic analyses in zebrafish. Similar to tetrapod epithelial appendages, components of major signaling pathways are expressed at early stages of scale development, including Hh^9^, Bmp^8^, Eda/Edar^8^, and Fgf^15^. Postembryonic mutagenesis screens for mineralized tissue defects have been particularly informative in revealing the molecular nature of scale development in zebrafish. Zebrafish harboring mutations in both *eda* and *edar* lack scales, which can be traced to a failure in scale placode formation^8^. A similar defect was documented in zebrafish homozygous for mutations in *fgfr1a*^15^. Further, evolved scale loss in both domesticated carp and the cyprinid genus *Phoxinellus* was also found to be due to mutations in paralogs of the *fgfr1* gene^15,16^. These insights underscore roles for these signal transduction pathways in early scale formation. They say less about whether these pathways also play a role in scale growth or differential scale shape.

**Fig. 4:**
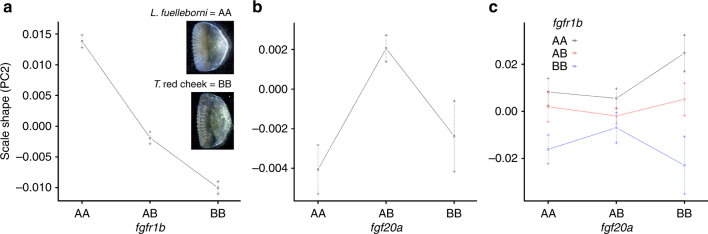
Epistasis between markers associated with *fgfr1b* and *fgf20a*. In all graphs, *Labeotropheus fuelleborni* possess the A allele, and *T*. red cheek possess the B allele. Thus, F_2_ animals denoted AA have inherited two *L. fuelleborni* alleles. **a** At the *fgfr1b* locus there is a strong association between genotype and scale phenotype. **b** At the *fgf20a* locus there is no such association. **c** When grouped by genotype at *fgf20a*, the genotype-by-phenotype association at *fgfr1b* is especially robust. The association is most pronounced in animals that are homozygous for the B allele at *fgf20a*. Bars indicate standard errors of mean phenotypic values for each genotype

Previous work in our lab has shown that deficiency in *fgf20a* results in aberrant mineralized tissue development in zebrafish, including scales^42^. This Fgf ligand was also implicated in evolved scale loss in *Phoxinellus*, and an epistatic interaction between *fgf20a* and *fgfr1a* was confirmed for scale development in zebrafish^16^. While an *fgfr1* paralog was implicated in regulating scale shape in our cross, *fgf20* was not (Fig. 4a, b). However, given the recent observations in zebrafish^15^ and *Phoxinellus*^16^ we sought to test the hypothesis that an epistatic interaction between these loci might influence cichlid scale shape. Notably, a strong interaction was observed for scale 3 PC2, whereby the genotypic effects on scale shape at *fgfr1b* were exaggerated in animals that were also homozygous for the *T*. red cheek allele at *fgf20a* (Fig. 4c). The observation that these loci interact during (1) scale development in zebrafish, (2) scale loss in *Phoxinellus*, and (3) scale shape in cichlids suggests that this in an evolutionarily conserved and important genetic interaction during fish scale development.

### Distinctly shaped scales differentially express fgfr1b

**Fig. 5:**
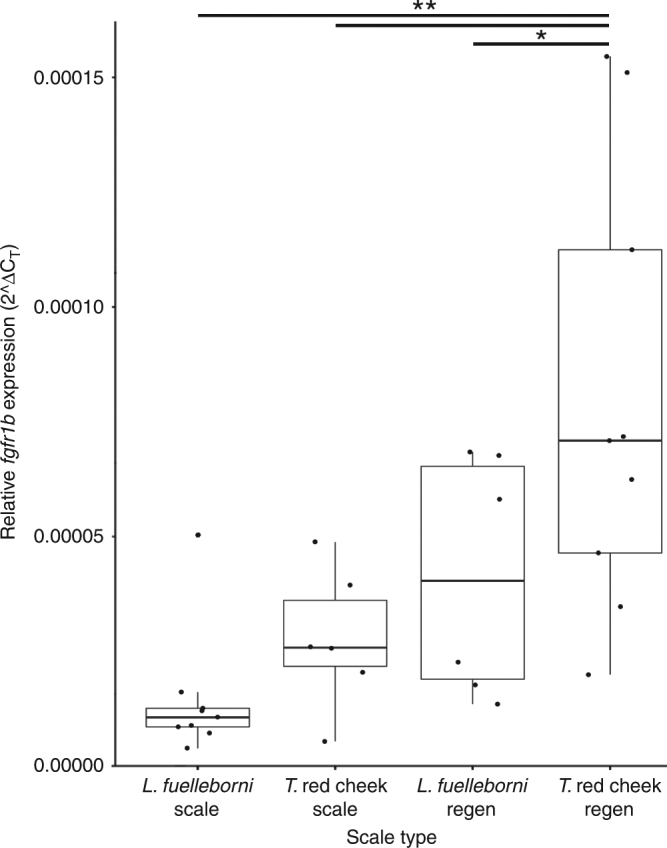
Quantitative rtPCR results for scale tissues. Box and whisker plot showing expression levels relative to the housekeeping gene, *beta actin*. All data points are shown as black dots. Error bars extend to the maximum and minimum values for each group, not including outliers. The center of each box depicts the median, and the upper and lower hinges correspond to the third and first quartiles, respectively. Relative expression is calculated via the comparative C_T_ method. Along the x-axis, species names followed by “scale” indicates expression in tissue around normally growing scales. Species names followed by “regen” indicates expression in scales after one week of regeneration. Asterisks indicate significance at the *p* < 0.05 (*) and *p* < 0.01 (**) levels

A key difference between the scales of *L. fuelleborni* and *T*. red cheek is height along the dorsal-ventral axis, and several QTL that influence relative scale height map to an interval that contains *fgfr1b*. We therefore sought to assess whether scales with different shapes express different amounts of *fgfr1b* transcripts. For this analysis, we took advantage of two developmental attributes of fishes: (1) indeterminate growth of mineralized tissues, and (2) ability to regenerate scales. Specifically, we compared expression levels in adult *L. fuelleborni* (*n* = 3) and *T*. red cheek (*n* = 3) scales under both normal growing and regenerative conditions. At day 0, RNA was extracted from six scales collect from two regions of the flank (i.e., regions 3 and 5) for each fish (i.e., *n* = 3 scales at each region, *n* = 6 scales total). At day 7, regenerating scales were collected from the same regions (i.e., *n* = 6 scales) for RNA extraction. At this stage scales were approximately 3/4 the size of fully regenerated scales. cDNA was synthesized and *fgfr1b* levels were compared between stages and species via quantitative rtPCR. For both species, transcript abundance was higher in regenerative versus normal scales (Fig. 5). In addition, the species with taller scales along the dorsal-ventral axis, *T*. red cheek, exhibited higher *fgfr1b* levels in both normal growing and regenerative scales. These data suggest that greater *fgfr1b* expression is associated with expanded scale growth in the dorsal-ventral dimension.

### Modulation of Fgf signaling mimics natural scale shape variation

**Fig. 6:**
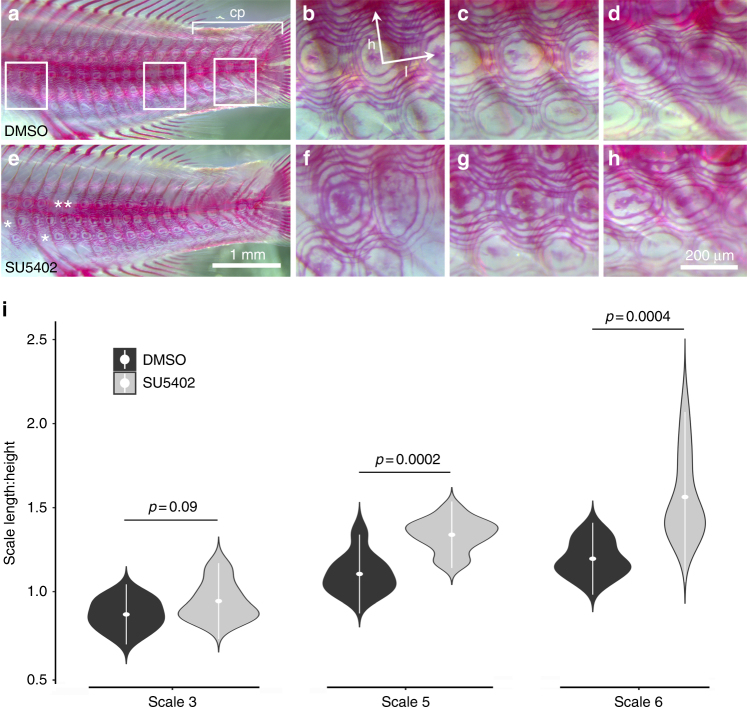
Treatment with the Fgfr inhibitor, SU5402, influences scale shape in cichlids. **a** Image of the lateral flank of a 3 weeks DMSO carrier control cichlid larvae (*T*. red cheek). Boxes indicate the three zoomed-in regions depicted in **b**–**d** from left to right. These regions roughly correspond to scale regions 3, 5, and 6 in previous analyses. The caudal peduncle (cp) region is indicated by the bracket. The arrows in **b** indicate the measures that were taken to calculate length (h) over height (l) ratios (l:h) used to compare experimental and control fish. (**e**–**h**) Similar views of the scales on larvae treated with 2.5 μM of SU5402 for 14 h at 2 weeks post fertilization. Asterisks in **e** mark a few regions where scales failed to develop due to SU5402 treatment. Scale bar for **a** and **e** is shown in **e**. Scale bar for **b**–**d** and **f**–**h** is in **h**. **i** Violin plots showing the differences in scale shape (i.e., length:height ratio) between DMSO carrier (*n* = 9) and SU5402 (*n* = 12) treated fish for scales 3, 5, and 6. Mean value and standard deviations are in white. *P*-values calculated with *t*-tests

Fgfs are known to influence early scale patterning^15^, but roles for later growth and shape are unknown. Results from our QTL and qPCR analyses implicated *fgfr1b* in shaping cichlid scales; we therefore chose to functionally test the role for Fgf signaling in differential scale development. Based on qPCR data (Fig. 5), our prediction was that knock-down of Fgf signaling will reduce dorsal-ventral scale height. To test this prediction, we dosed *T.* red cheek larvae with 2.5 μM of the Fgfr antagonist, SU5402, for 12 h at 2 weeks post-fertilization. Importantly, the timing of this treatment allowed us to assess the role for Fgf signaling in both placode specification and scale growth. At the time of treatment, cichlid larvae possess several rows of midline scales extending anteriorly from the caudal peduncle (cp in Fig. 6a), in which we could assess the role of Fgf signaling in scale shape. From these established scales, scale development continues anteriorly as well as dorsal-ventrally until the body is covered^9^. SU5402 treatment allowed us to confirm the known role of Fgf signaling in scale patterning (i.e., placode specification) in these newly developing scales, which served as an internal control for our treatment regime. After treatment, animals were washed several times and reared for an additional week in system water.

Roles for Fgf signaling in early scale/placode pattering could be confirmed by assessing the degree to which SU5402 treatment blocked scale formation (Fig. 6). Additional roles in scale shape/growth were determined by analyzing scales that were well-formed pre-treatment. As expected, treatment with SU5402 blocked scale development in regions of the body where scales had not yet formed (asterisks in Fig. 6e). Notably, scale shape was also affected in regions where scale development was already well underway upon treatment. Qualitatively, SU5402 led to aberrant mineralization of scales (Fig. 6f–h). Quantitatively, fish treated with SU5402 developed scales that were significantly more elliptical in shape compared to carrier control animals (i.e., longer along the anterior-posterior axis, shorter along the dorsal-ventral axis, Fig. 6i–k). Taken together these data show that in addition to placode initiation, Fgf signaling is necessary for proper scale growth and shape, and supports the hypothesis that variation at *fgfr1b* contributes to scale shape variation, especially relative scale height, at the QTL on LG7.

## Discussion

Fish scales are diverse, and this diversity has been the focus of an array of biological questions, including species identification^43,44^, taxonomic relationships^45,46^, characterization of extinct taxa^47,48^, age and growth rates^49,50^, fluid mechanics^51,52^, and biomimetics^53–55^ to name a few. Despite this long history, the ecological significance of variation in scale shape remains poorly understood. Whereas differences in scale size often have a direct functional significance (e.g., small scales for increased hydrodynamics, large scales for protection), connecting more subtle aspects of scale morphology to species performance and fitness has proven more difficult.

Here we show that closely related, and ecologically similar species of cichlids exhibit measureable differences in scale morphology. Notably, *L. fuelleborni* and *T*. red cheek do not differ in scale number along the flank. Both species develop similar numbers of body scales in the anterior-posterior and dorsal-ventral dimensions (Supplementary Figs. 9–10). Thus, patterning at the placode stage appears to be conserved between these species. Rather, differences appear to be limited to scale shape. In addition, the consistency of allele effects for certain scale traits suggest that this variation may be maintained by divergent selection^19,33,56^. Nevertheless, the adaptive value for cichlid scale shape variation remains a matter of speculation.

The most conspicuous differences between *L. fuelleborni* and *T*. red cheek scales pertain to their relative height. *L. fuelleborni* possess generally more cycloid-shaped scales across the body, whereas *T*. red cheek scales are more oblong in the dorsal-ventral dimension. This difference in scale shape may facilitate the alternate modes of feeding in the two species. While both occupy the upper reaches of the near-shore rocky habitat and forage on attached algae, the mechanism of food collection is very different. *L. fuelleborni* possess wide, heavily fortified jaws that are used to crop large mouthfuls of algae while swimming parallel to the substrate. Body movement in *L. fuelleborni* during feeding, especially lateral bending, is minimal^57^. Alternatively, *T*. red cheek forages by hovering over algal beds, grasping strands of algae with their narrow beak-shaped mouths and jerking their bodies to one side^26,27^. Shorter scales in the anterior–posterior dimension might facilitate this twisting motion.

*L. fuelleborni* and *T*. red cheek also differ in radii number and length, with *T*. red cheek possessing more and longer radii on average than *L. fuelleborni*. These structures constitute gaps in the mineralized portion of scales that are anchored in the dermis. Radii are postulated to decrease bending stiffness and increase flexibility of scales without compromising their protective function (reviewed in ref. ^14^). Like most Lake Malawi cichlids, *L. fuelleborni* and *T*. red cheek males are highly territorial and will engage in competitive interactions that include flank nipping, an action that often results in the dislodging of body scales. Thus, developing well-fortified scales is likely important for both species. The occurrence of a greater number of radii in *T*. red cheek may therefore reflect a balance between body bending during foraging and protection during competitive interactions.

A balance between alternate functional demands in feeding and protection in *T*. red cheek might also underlie the observation that this species exhibits greater disparity in scale shape across the body relative to *L. fuelleborni*. That is, *T*. red cheek may possess a wider variety of scale shapes across the body to accommodate regions involved in either bending of the body during feeding, or necessary for providing protection during competitive interactions. This disparity in scale shape would be interesting to examine in a greater number of species and across additional regions of the body (e.g., dorsal-ventral). From an ecological perspective, differences in scale shape disparity between species could reveal insights into how species partition niche-space. For instance, differences in 3D scale shape within blue gill sunfish, have been postulated to correlate with distinct functional demands (i.e. fluid mechanics) experienced by different regions of the body^14^. If true, one might predict a higher degree of scale shape disparity in species that utilize speed and/or maneuverability during prey capture (e.g., twisting in *T*. red cheek), and a lower degree of disparity in species that utilize a less dynamic mode of prey capture (e.g., *L. fuelleborni*).

From an evolutionary perspective, such differences between species offer a unique opportunity to gain insights into the evolvability of serially homologous structures. This is relevant not only to scales, but to other epithelial appendages as well. For example, considerable variation exists in tooth shape disparity among mammals, from near homodonty in seals and ontodocete whales, to the extreme elaboration of a single tooth class in elephants, narwhals, and walruses. While some molecular work has begun to address the basis for tooth shape differences with the same jaw (e.g. refs. ^58,59^), we are largely ignorant about how such differences evolve at the genetic/genomic level. In addition, birds and mammals can exhibit dramatic variation in the distribution of feathers and hair, respectively, across the body, and some progress has been made in identifying the genetic variants that mediated such variation^60,61^. Despite these examples, the molecular mechanisms that facilitate disparity among serially homologous epithelial appendages remain largely elusive. Given that the differential evolution of serial homologs represents an important mechanism of morphological diversification in metazoans in general^62^, this remains an important question in the field. Fish offer an ideal model to explore such questions as they offer an excellent balance between experimental utility (e.g., transgenic resources in zebrafish, and genomes for many species) and evolutionary richness (e.g., many well characterized adaptive radiations). We therefore maintain that investigations in fish scales have the potential to address important questions with respect to the development and evolution of serially homologous structures.

## Conclusions

Understanding the molecular mechanisms that contribute to variation in complex morphologies represents an important ongoing challenge in the field^63^. We have argued previously that a comparative approach in non-traditional models holds much promise for this pursuit^64^. Here we illustrate this idea using variation in scale morphology as a model. Insights from this study underscore several keys points. First, subtle variation in scale shape, including disparity across the body, has a tractable genetic basis. Second, the same major signaling pathway is involved in both the early patterning and later shaping of the same phenotype. Finally, the integration of genetic data sets across traits and experiments has the potential to yield broad insights into the roles for pleiotropy in organismic adaptation. The QTL hotspot for scale shape that includes the *fgfr1b* locus co-localizes with a QTL for skull shape in this same cross^20^. This raises the possibility that the evolutionary response for distinct mineralized tissues is influenced by a degree of genetic integration (e.g., “genetic lines of least resistance”^65^). To paraphrase^66^, and recalling^67^, nothing in evolution makes sense except in the light of integration. Traits rarely evolve in isolation. Rather, adaptive suites of traits seem to respond to selection in a coordinated manner. Piecing together the genetic architecture of trait variation is a tedious task, but one that is necessary to understand the proximate mechanisms that promote the evolution and co-evolution of phenotypes. Accomplishing this in traits that are representative of more general traits classes (e.g., pigmentation, neural, mineralized tissue, epithelial appendages) holds promise in producing results that may be generalized to other systems and phenotypes. In doing so, this accumulated knowledge will help to achieve a primary goal in evolutionary biology—i.e., to identify the factors that contribute to the origins and maintenance of biodiversity.

## Methods

### Quantification of scale shape variation

Twelve individuals from each parental species, *L. fuelleborni* and *T.* red cheek, were phenotyped for this study, as well as 256 F_2_ hybrids from their cross. All animals were reared and sacrificed according to the University of Massachusetts Institutional Animal Care and Use Committee (IACUC). A mix of females and males were used (e.g., 52.7% female in the F_2_ hybrids), and sex assignments for the F_2_ hybrids can be found in data available on dryad (see Data availability section). All animals were sexually mature adults, aged 9–12 months. In fishes, standard length is a standard proxy for age/stage. All parental and F_2_ hybrid animals were between 5.0–9.7 cm standard length, with full data available on dryad. As noted below, all measurements were normalized to standard length. For each animal, scales were taken from six distinct regions along the midline of the body, spanning from just posterior of the opercle to the caudal peduncle (Fig. 1b). Each scale was imaged with a scale bar using a Leica DFC450 camera mounted to a Leica MF15 stereomicroscope. From these images, various measurements were taken using the ImageJ software program (Fig. 1a), including the length of the scale in the anterior–posterior dimension, the height of the scale (i.e., dorsal–ventral), the length of the radii, the height of the anterior margin of the radii, the height of the posterior margin of the radii, the angle at which the outer most radii extended to the focus, and the total number of radii present. To remove the effects of allometry on scale shape, all measurements were converted into residual data by normalizing to standard length.

Shape variation among scales was also quantified via geometric morphometric shape analysis. Homologous anatomical landmarks defined the anterior, posterior, dorsal, and ventral edges of the scale as well as the anterior margins of the radii (Fig. 1a). The position of these landmarks was collected from photos using tpsDig2 software^68^. The program tpsRelw^69^ was used to conduct Procrustes superimposition of landmarks, which removed variation due to size, rotation, and position, leaving only variation between scales due to shape. Allometry was also removed from the data via a multiple regression of shape on geometric centroid size to generate landmark data sets based on residuals for further analysis. Variation in scale shape was analyzed for each of the six scales individually in both parentals and F_2_ hybrids. All six scales were also analyzed together; the degree of shape disparity among all six scales from an individual was measured using the morphol.disparity function in the geomorph package in R^70^ with 2000 iterations. This function estimates disparity as a single quantitative measure based on the Procrustes variance of all scales for each individual.

### Pedigree, RAD-seq, and linkage map construction

A single *L. fuelleborni* female was crossed to a single *T*. red cheek male, creating a single F_1_ family, which was subsequently incrossed to produce a F_2_ hybrid mapping population. Genomic DNA was extracted from pectoral fin tissue using DNeasy blood and tissue kits (Qiagen Inc. CA, USA), digested with the restriction enzyme *SbfI* and processed into RAD libraries following^71^. Barcoded, processed and purified DNA from 268 F_2_ as well as 20 wild-caught *L. fuelleborni* from Makanjila Point and 20 wild-caught *T*. red cheek from Chizumulu Island was sequenced using an Illumina HiSeq 2000 (Illumina, San Diego, CA) and single-read (100 bp) sequencing chemistry. Sequencing and bioinformatics followed^71^ and are described in greater detail in ref. ^35^. Briefly, Bowtie^72^ was used to align reads to the Lake Malawi cichlid reference genome (i.e., *Metriaclima zebra* v0), and SAMTOOLS was used for SNP calling. In total, >42 K SNPs were identified across all samples. Most represented rare variants, and the data set was filtered for deviations from Mendelian segregation. We also filtered our SNPs based on population-level signatures of divergence. Specifically, the per-locus estimates of F statistics (F_ST_, F_STP_ and F_IS_) were calculated following^73^ using the R package HIERFSTAT (R core team). We considered loci to exhibit a signature of divergence when F_ST_ > 0.6, an empirical threshold for divergence between cichlid genera^34^. This resulted in 1395 loci available for linkage map construction. Most of those markers represented SNPs with outlier F_ST_ values, but ~200 were also included to span physical scaffolds that did not contain outlier SNPs from this analysis. A complete list of SNPs with outlier F_ST_ values is provided elsewhere^35^. Linkage map construction followed methods contained within the R/qtl package^74^, and are presented in detail specific to our cross within another work^35^. The resulting map contained 948 loci consisting of 25 linkage groups (LGs), 24 of which had between 13 and 76 markers (the 25th LG contained only two loci and was excluded from the current analysis). The total map size was 1474.9 cM. Linkage groups were numbered based on ref. ^75^.

### QTL analysis

QTL mapping followed the multiple-QTL mapping (MQM) method using routines in R/qtl presented by ref. ^76^. The first step in this process is a liberal scan for unlinked QTL using the onescan function in R/qtl^77^. This analysis provides a list of putative QTL intervals (e.g., generally approaching or above a LOD of 3.0) that were used to build more rigorous models. Specifically, MQM scans use these loci as potential cofactors, which are verified by backward elimination. The inclusion of unlinked cofactors in the final model helps to more accurately detect and assess the effects of QTL^78^. Significant QTL reported here possess peak LOD scores greater than the 95% confidence threshold, determined by 1000 permutations.

### Quantitative real-time PCR

Quantitative PCR was used to measure the expression of *fgfr1b* in the scales of adult fish (7.0–9.0 cm standard length) *L. fuelleborni* (*n* = 3, one male, two female), and *T*. red cheek (*n* = 3, one male, two female). Primer sequences were: Forward (5ʹ-3ʹ) ACTGCCTCCTGCTGGTTCT, Reverse (5ʹ-3ʹ) GATTCGTGGTCCTTCCTCA. Tissue was taken from both normally growing and regenerating scales. Specifically, on the left flank of each fish three scales were removed from regions 3 and 5 (*n* = 6 scales in total), combined in one tube, and stored in Trizol at −20 °C. After 1 week, regenerating scales were removed from the same regions (*n* = 6 regenerating scales for each fish). Next, RNA was isolated from homogenized scale tissue via phenol chloroform extraction, and standardized prior to reverse transcription. Finally, levels of gene expression were measured using SYBR Green chemistry (Power SYBR Green Master Mix), and relative quantification (compared to *beta actin*) was analyzed using the comparative CT method^79^.

### Small molecule manipulations

A 5 mM stock solution of SU5402 (Sigma-Alrich) was prepared using Dimethyl Sulfoxide (DMSO, Sigma-Alrich) as a solvent. Because we wanted to assay roles for Fgf signaling in scale shape, not patterning, *T*. red cheek larvae (*n* = 21) were raised to 2 weeks post fertilization. It is not possible to determine sex of these fish larvae. Several rows of midline scale are well formed by this stage, as confirmed by labeling mineralized tissues pre-treatment with the fluorochrome calcein green (Sigma-Alrich). Scales that were well formed at the time of SU5402 addition were the ones that were analyzed post-treatment. A working stock of 2.5 μM was made in system water. A carrier control solution was also made by adding the same volume of DMSO to system water. This concentration of SU5402 is based on previously published work on small molecule manipulations in cichlids^80^, as well as on our own experiences. The dose was titrated to allow larvae to survive long-term exposure without the development of gross anomalies. For instance, a 5 μM dose consistently resulted in a high frequency of animals developing generalized developmental defects. Animals were collected from two crosses, to account for family effects. In total, 12 animals were used for SU5402 treatments, and nine were used for carrier controls. To mitigate the chances that altered scale shape might arise via compensatory growth, we only measured scales that were surrounded by other scales. For all experiments, fry were incubated for 14 h in Erlenmeyer flasks at 28.5 °C. After treatment, experimental and control animals were rinsed several times in fresh system water, and raised for an additional week, at which point they were sacrificed accorded to a protocol approved by the UMass IACUC, fixed, and cleared and stained.

### Alizarin red staining of mineralized tissue

Clearing and staining followed^81^. Throughout the process, care was taken to avoid loss of scales and collection tubes were monitored for dislodged scales. Specimens were fixed in 4% paraformaldehyde for at least 24 h at room temperature (up to 1 week at 4 °C). After fixation, animals were washed several times (3 × 1 h) in phosphate buffered solution (PBS), and viscera were removed with fine forceps. Samples were transferred to a 0.5% potassium hydroxide solution with enough aqueous (0.5%) alizarin red (Sigma-Alrich) added to turn the solution a deep, nearly opaque, purple. Specimens were stained overnight in this solution, and then washed 1 × 10 min in 0.5% KOH, and then 2 × 10 min in PBS. Pigment was then removed by adding several drops of 30% hydrogen peroxide to the PBS washes. Tissue was cleared via a brief typsin digest (30 mL saturated aqueous sodium borate + 30 mL distilled H_2_O + 1 g Trypsin powder (Sigma-Alrich)). To prevent dislodging of scales, fish were only gently and briefly digested. The reaction was stopped once the skull sutures on the top of the head were clearly visible. Specimens were washed several times in PBS, taken through a graded glycerol series, and preserved in 80% glycerol. Images were taken using a Leica DFC450 camera mounted to a Leica MF15 stereomicroscope. Scale measurements were obtained in ImageJ, and statistical analyses were done in R.

### Data availability

The datasets generated during and/or analysed during the current study are available on dryad, 10.5061/dryad.35cp405 ^82^. Genotypic data for QTL mapping is also available on dryad, 10.5061/dryad.7sr73 ^83^.

## Electronic supplementary material

Supplementary Information
