# Supplementary material for: Genetic analyses in Lake Malawi cichlids identify new roles for Fgf signaling in scale shape variation
Source: Commun Biol. 2018 May 31;1:55. doi: 10.1038/s42003-018-0060-4 (PMC6123627; doi:10.1038/s42003-018-0060-4)
Supplement: Supplementary file 1 — Supplementary Information [file 42003_2018_60_MOESM1_ESM.pdf]

Supplementary Figures

Supplementary Figure 1: Violin plots for dorsal to ventral length measurements in parental species (n=12 each), as well as their F_2 hybrids (n=50, randomly chosen). All values are residuals against standard length. Mean values and standard deviations are in black. Statistical significance between parental species were assessed via ANOVA in R. Significance is represented as follows: '***' <0.001; '**' <0.01; '*' <0.05; '.' <0.1; ' ' >0.1. *L. fuelleborni* is in yellow, F_2 hybrids are in gray, and *T.* red cheek is in blue.

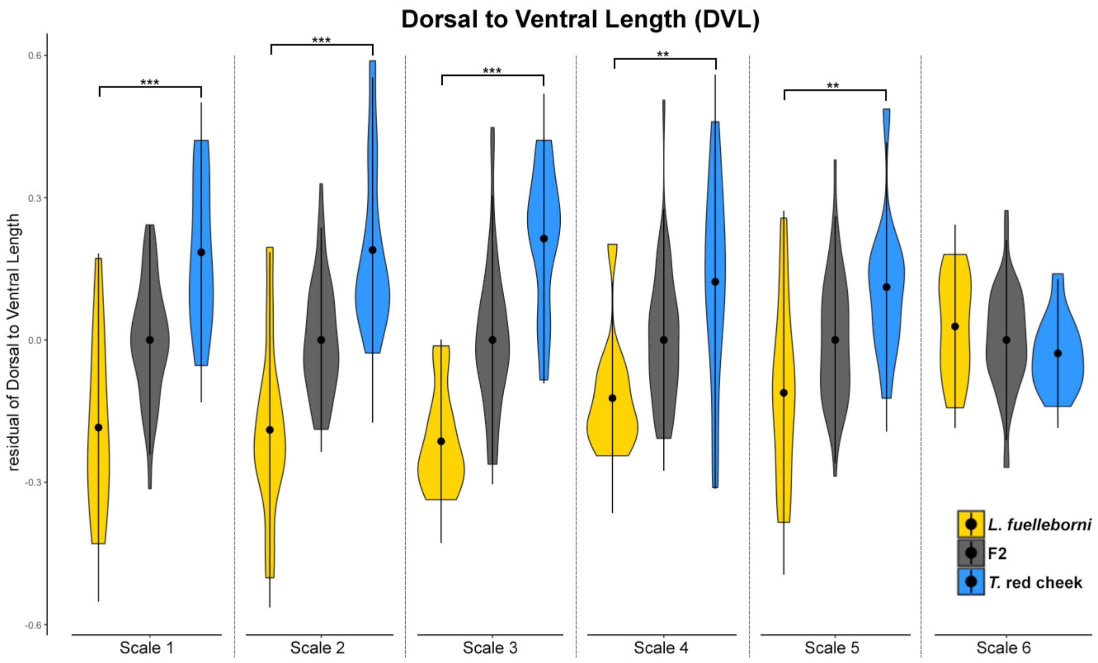

Supplementary Figure 2: Violin plots for anterior to posterior length measurements in parental species (n=12 each), as well as their F_2 hybrids (n=50, randomly chosen). All values are residuals against standard length. Mean values and standard deviations are in black. Statistical significance between parental species were assessed via ANOVA in R. Significance is represented as follows: '***' <0.001; '**' <0.01; '*' <0.05; '.' <0.1; ' ' >0.1. *L. fuelleborni* is in yellow, F_2 hybrids are in gray, and *T.* red cheek is in blue.

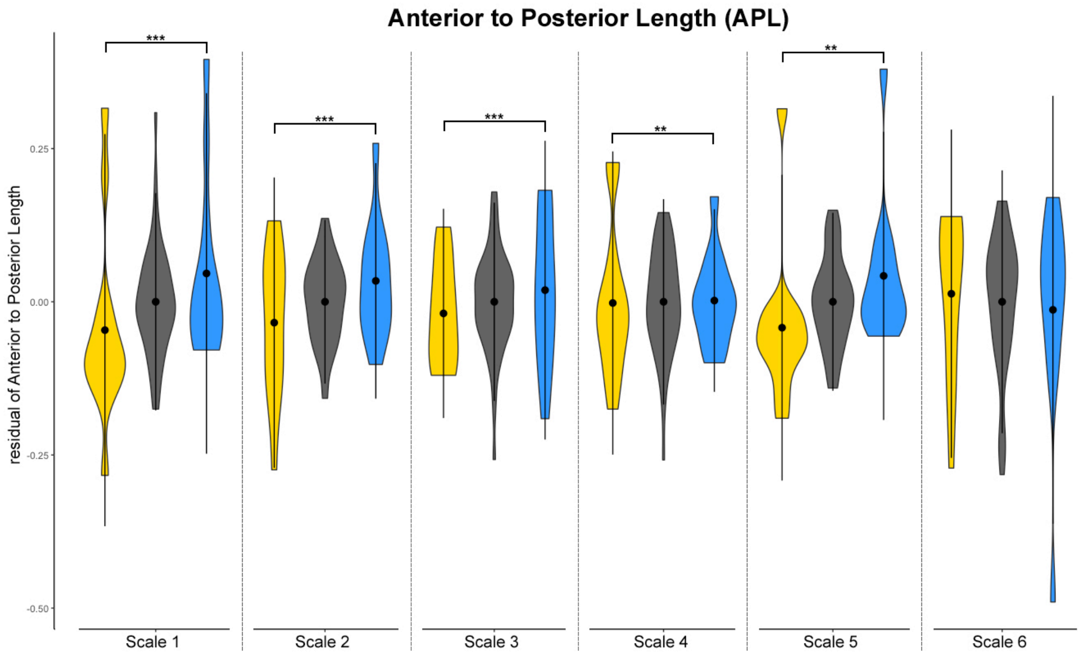

Supplementary Figure 3: Violin plots for the ratio of dorsal-ventral length to anterior-posterior length in parental species (n=12 each), as well as their F_2 hybrids (n=50, randomly chosen). All values are residuals against standard length. Mean values and standard deviations are in black. Statistical significance between parental species were assessed via ANOVA in R. Significance is represented as follows: '***' <0.001; '**' <0.01; '*' <0.05; '.' <0.1; ' ' >0.1. *L. fuelleborni* is in yellow, F_2 hybrids are in gray, and *T.* red cheek is in blue.

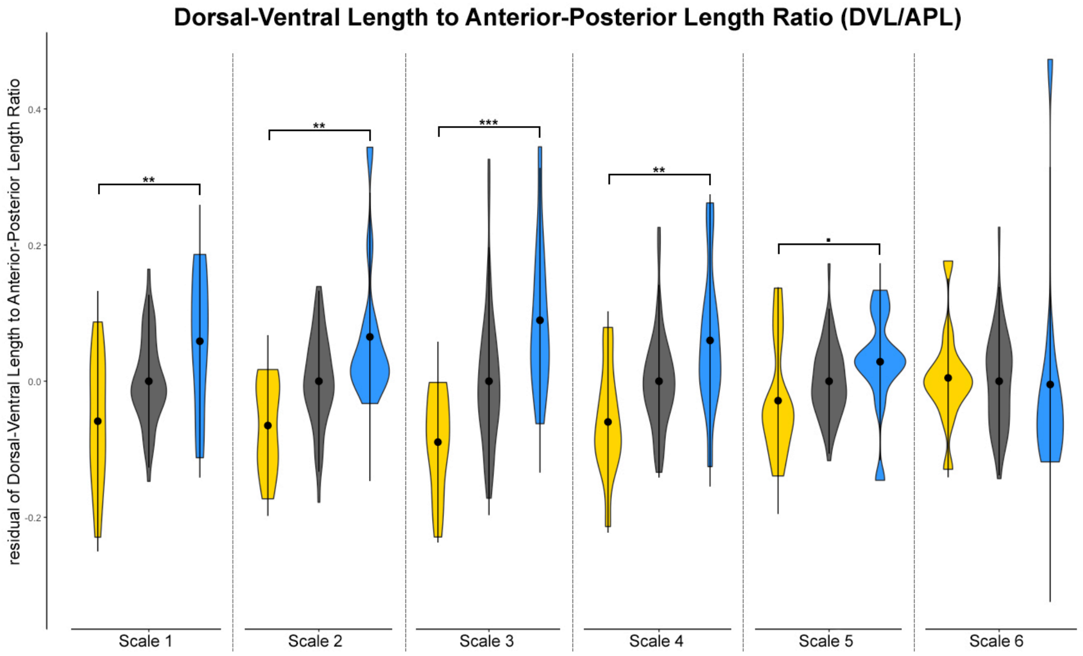

Supplementary Figure 4: Violin plots for measurements of radii length in parental species (n=12 each), as well as their F_2 hybrids (n=50, randomly chosen). All values are residuals against standard length. Mean values and standard deviations are in black. Statistical significance between parental species were assessed via ANOVA in R. Significance is represented as follows: '***' <0.001; '**' <0.01; '*' <0.05; '.' <0.1; ' ' >0.1. *L. fuelleborni* is in yellow, F_2 hybrids are in gray, and *T.* red cheek is in blue.

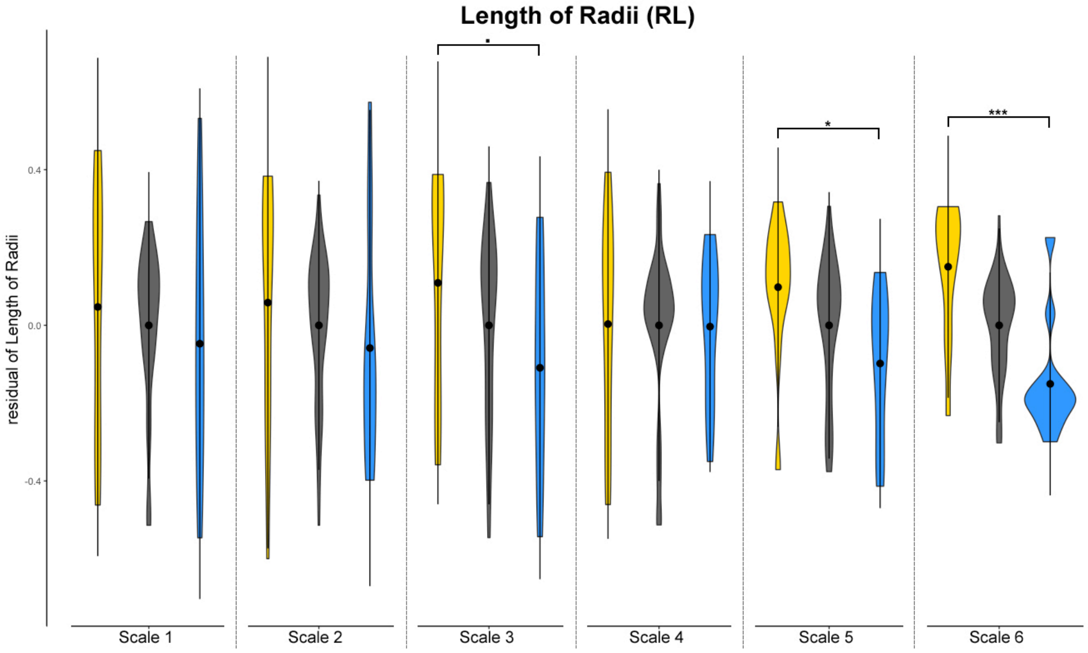

Supplementary Figure 5: Violin plots for length of anterior margin of radii measurements in parental species (n=12 each), as well as their F_2 hybrids (n=50, randomly chosen). All values are residuals against standard length. Mean values and standard deviations are in black. Statistical significance between parental species were assessed via ANOVA in R. Significance is represented as follows: '***' <0.001; '**' <0.01; '*' <0.05; '.' <0.1; ' ' >0.1. *L. fuelleborni* is in yellow, F_2 hybrids are in gray, and *T.* red cheek is in blue.

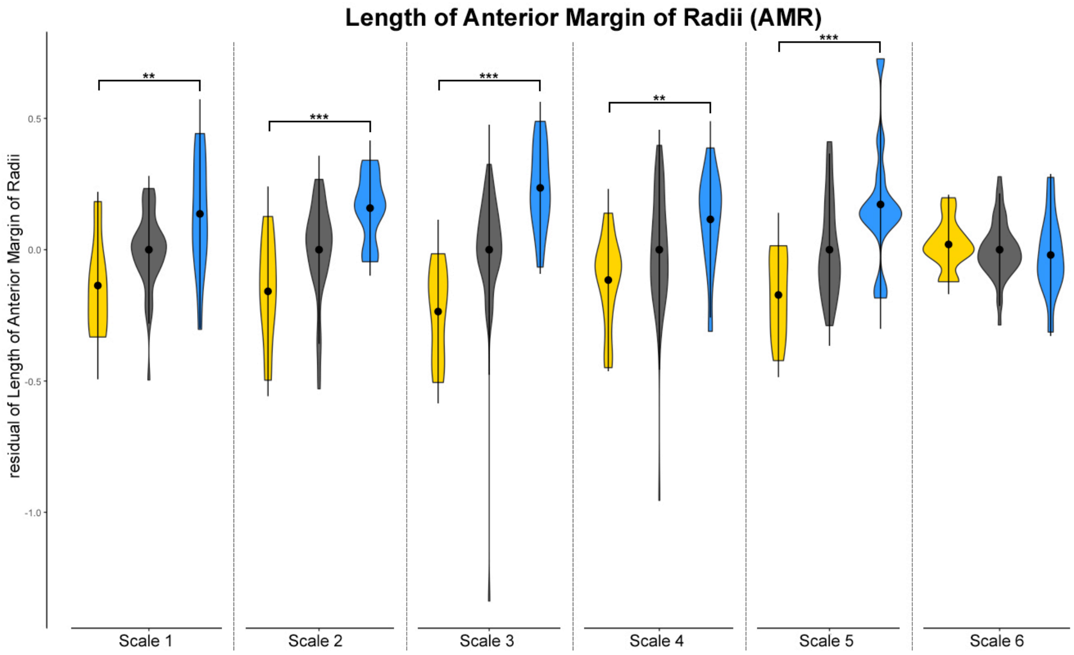

Supplementary Figure 6: Violin plots for length of posterior margin of radii measurements in parental species (n=12 each), as well as their F_2 hybrids (n=50, randomly chosen). All values are residuals against standard length. Mean values and standard deviations are in black. Statistical significance between parental species were assessed via ANOVA in R. Significance is represented as follows: '***' <0.001; '**' <0.01; '*' <0.05; '.' <0.1; ' ' >0.1. *L. fuelleborni* is in yellow, F_2 hybrids are in gray, and *T.* red cheek is in blue.

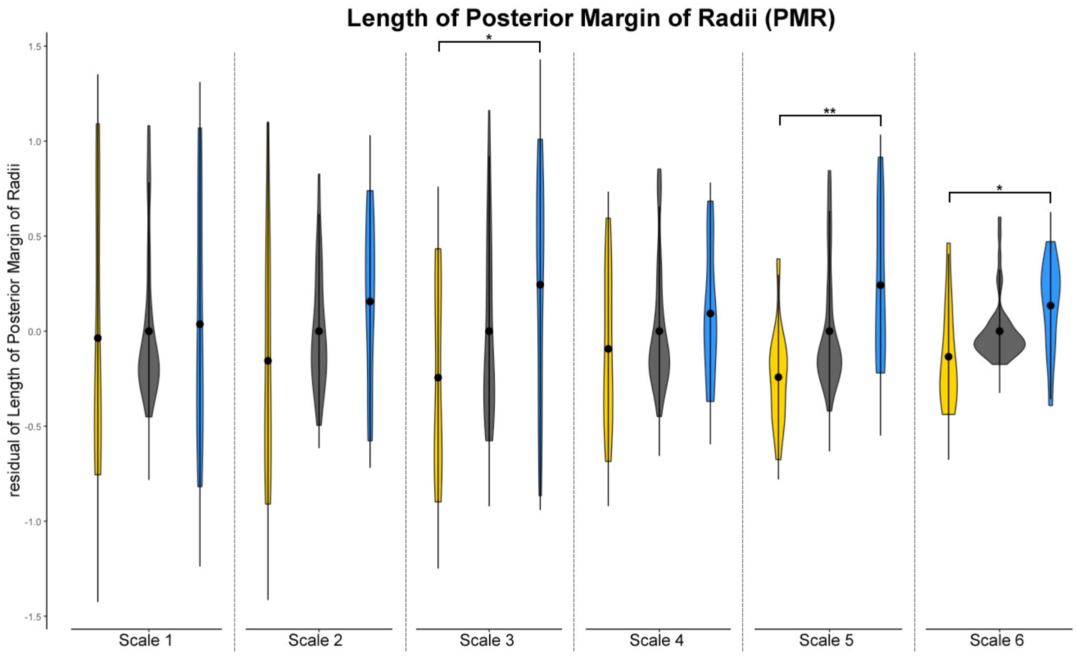

Supplementary Figure 7: Violin plots for angle of radii to focus measurements in parental species (n=12 each), as well as their F_2 hybrids (n=50, randomly chosen). All values are residuals against standard length. Mean values and standard deviations are in black. Statistical significance between parental species were assessed via ANOVA in R. Significance is represented as follows: '***' <0.001; '**' <0.01; '*' <0.05; '.' <0.1; ' ' >0.1. *L. fuelleborni* is in yellow, F_2 hybrids are in gray, and *T.* red cheek is in blue.

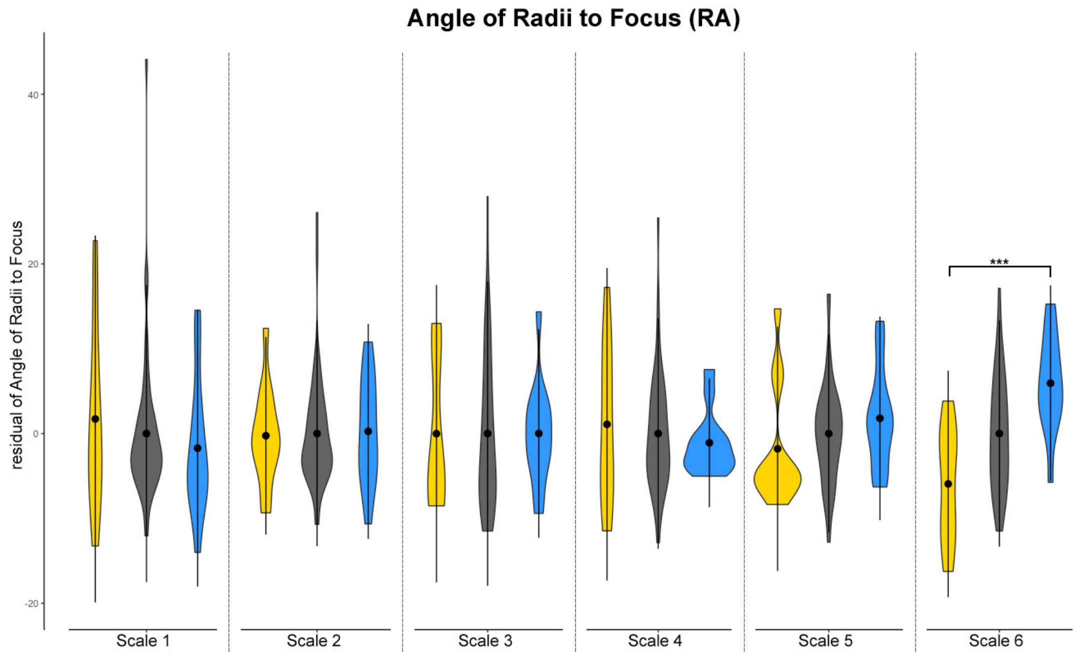

Supplementary Figure 8: Violin plots for number of radii in parental species (n=12 each), as well as their F_2 hybrids (n=50, randomly chosen). All values are residuals against standard length. Mean values and standard deviations are in black. Statistical significance between parental species were assessed via ANOVA in R. Significance is represented as follows: '***' <0.001; '**' <0.01; '*' <0.05; '.' <0.1; ' ' >0.1. *L. fuelleborni* is in yellow, F_2 hybrids are in gray, and *T.* red cheek is in blue.

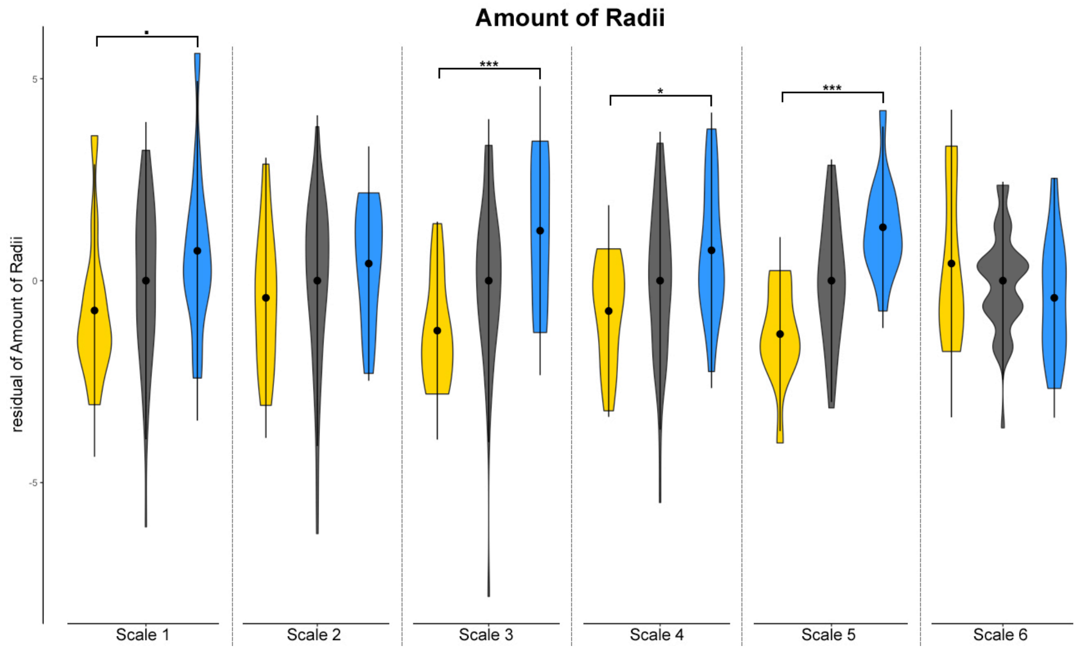

Supplementary Figure 9: Violin plots for number of scales in anterior to posterior axis in parental species (n=12 each), as well as their F_2 hybrids (n=50, randomly chosen). All values are residuals against standard length. Mean values and standard deviations are in black. There was no statistical significance between parental species as assessed via ANOVA in R. *L. fuelleborni* is in yellow, F_2 hybrids are in gray, and *T.* red cheek is in blue.

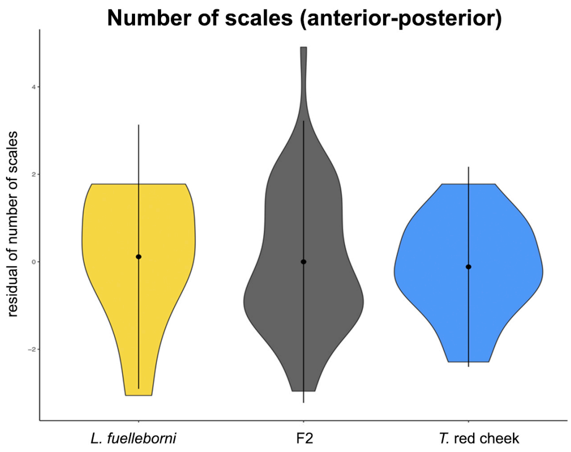

Supplementary Figure 10: Plots for number of scales in dorsal to ventral axis in parental species (n=12 each), as well as their F_2 hybrids (n=50, randomly chosen). Due to lack of variation, data is not presented as a violin plot, but as individual points. All values are residuals against standard length. Mean values are in black. There was no statistical significance between parental species as assessed via ANOVA in R. *L. fuelleborni* is in yellow, F_2 hybrids are in gray, and *T.* red cheek is in blue.

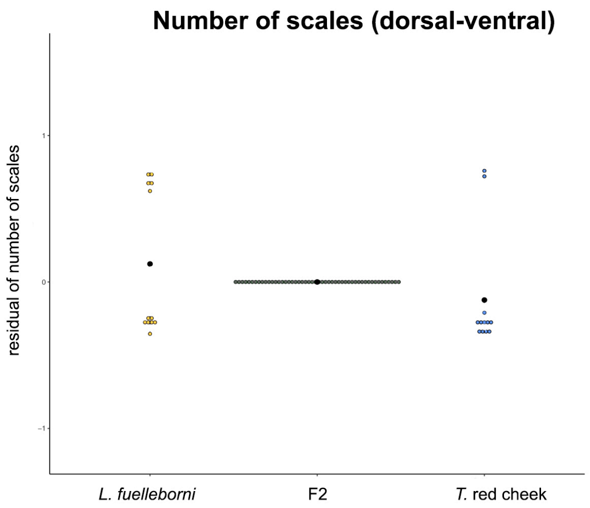

Supplementary Figure 11: Deformation grids for scale shape variation in parental species (i.e., *L. fuelleborni* and *T.* red cheek) as well as in the F_2. Results for scales 1-6 are depicted on the left for the parental species. Only PC1 is reported, as this was the axis that distinguished parental species (except for scale 6, which did not differ between species). Within each of these boxes the percent variance explained (PVE) is reported, as well as the p-value from a t-test comparing *L. fuelleborni* and *T.* red cheek values along each axis. To the right, results are reported for the F_2 for PCs 1-3. Only scales 3 and 5 were analyzed in the F_2, as these were the most divergent between parental species in terms of both geometric and linear morphometrics. PVEs are similarly reported for each PC in both scales. These data correspond to those reported in Table 1 in the main text.

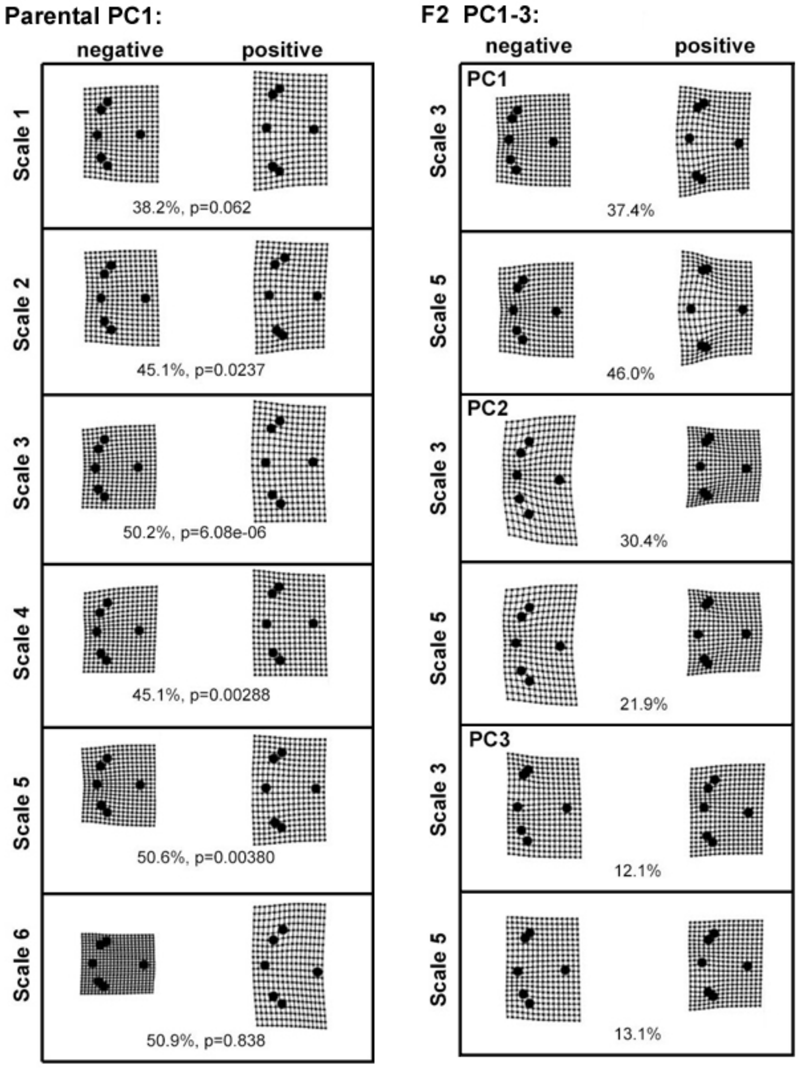

Supplementary Tables

Supplementary Table 1: List of quantitative trait loci (QTL) affecting scale shape variation. Trait and QTL names denoted with a "3" or "5" refer to scales 3 and 5, respectively. Abbreviations: Add, additive value; cM, centimorgan; Dom, dominance value; Effects, mean phenotypic value for each genotype; Lf, *L. fuelleborni*; LG, linkage group number; LOD, logarithm of the odds; PVE, percent variance explained; Trc, *T.* red cheek. All QTL were significant at the 0.05 genome-wide level (based on 1000 permutations), except those denoted †, which were suggestive at the 0.10 genome-wide level.

							Effects				
**Traits**	**QTL**	**LG**	**cM**	**QTL interval**	**LOD**	**PVE (%)**	Lf/Lf	Lf/Trc	Trc/Trc	**Add**	**Dom**
Radii Angle 3	3RA1	15	0	0.0-12.0	3.75	7.34	0.5095	-1.3100	-2.7279	1.6187	-0.2008
	3RA2	4	20	9.6-21.2	4.41	8.60	-1.1471	0.4495	-3.9704	1.4116	3.0083
	3RA3	9	5	0.0-13.7	4.60	8.95	-0.4676	0.2223	-5.3008	2.4166	3.1065
Anterior Margin of Radii 3	3AMR1	17	40	32.5-53.7	3.74	7.35	0.0555	-0.0120	-0.0035	0.0295	-0.0380
	3AMR2	5	45	34.9-50.9	3.44	6.77	-0.0317	0.0159	0.0514	-0.0415	0.0061
	3AMR3	18	0	0.0-1.5	5.54	10.67	-0.0875	0.0408	0.0314	-0.0595	0.0688
	3AMR4	19	10	2.5-21.6	3.75	7.36	0.0052	-0.0113	0.0621	-0.0284	-0.0450
Posterior Margin of Radii 3	3PMR1	7	0	0.0-14.6	4.68	9.10	-0.0561	-0.0382	-0.1283	0.0361	0.0540
	3PMR2	6	45	29.4-59.3	3.27†	6.45	-0.0175	-0.1431	0.0134	-0.0155	-0.1410
	3PMR3	15	15	5.0-26.8	4.47	8.71	0.0369	-0.1451	-0.0445	0.0407	-0.1413
	3PMR4	1	35	24.6-55.9	3.45	6.78	-0.0130	-0.1448	0.0331	-0.0231	-0.1548
Number of Radii 3	3NR1	18	5	0.0-12.8	5.00	9.69	-0.6920	0.4751	0.1887	-0.4404	0.7267
Dorsal-Ventral Length 5	5DVL1	7	45	33.7-57.9	5.04	9.76	-0.0398	-0.0106	0.0503	-0.0451	-0.0159
	5DVL2	12	25	13.9-47.1	3.70	7.27	-0.0284	0.0010	0.0505	-0.0392	-0.0100
Anterior-Posterior Length 5	5APL1	7	45	43.7-57.9	5.92	11.37	-0.0207	-0.0043	0.0367	-0.0287	-0.0123
	5APL2	21	35	13.5-45.0	4.74	9.21	0.0078	0.0104	-0.0094	0.0086	0.0112
Dorsal-Ventral to Anterior-Posterior Ratio 5	5DVAP1	6	60	29.4-64.4	5.66	10.89	-0.0054	-0.0105	0.0101	-0.0077	-0.0128
	5DVAP2	4	35	25.0-43.6	4.36	8.50	0.0007	0.0077	-0.0237	0.0122	0.0192
	5DVAP3	16	40	5.2-56.0	3.33†	6.55	0.0075	-0.0143	0.0079	-0.0002	-0.0219
	5DVAP4	2	15	8.1-33.8	3.66	7.19	-0.0368	0.0020	0.0121	-0.0244	0.0143
	5DVAP5	12	25	22.6-47.1	6.30	12.04	-0.0100	-0.0143	0.0253	-0.0177	-0.0220
Radii Length 5	5RL1	22	5	0.0-29.0	3.85	7.55	-0.0140	0.0724	-0.0853	0.0357	0.1220
	5RL2	14	10	9.8-16.8	6.01	11.53	-0.0465	0.0190	0.0005	-0.0235	0.0420
	5RL3	20	50	44.5-67.9	5.45	10.51	0.0463	0.0177	-0.0881	0.0672	0.0386
	5RL4	9	15	0.0-19.3	3.60	7.08	0.0431	0.0069	-0.0450	0.0440	0.0079
Radii Angle 5	5RA1	17	45	7.9-63.1	3.20†	6.32	0.0289	0.0252	1.0641	-0.5176	-0.5213
	5RA2	12	55	47.1-57.7	3.91	7.66	1.4209	1.0462	-1.9119	1.6664	1.2916
	5RA3	19	15	13.4-40.0	4.08	7.97	-0.7849	-0.0394	2.8132	-1.7991	-1.0536
Anterior Margin of Radii 5	5AMR1	17	40	32.5-47.3	3.76	7.38	0.0334	-0.0091	-0.0091	0.0213	-0.0213
	5AMR2	5	5	0.0-11.0	6.65	12.67	-0.0710	0.0330	0.0375	-0.0542	0.0497
	5AMR3	12	50	22.6-53.7	4.99	9.68	-0.0636	0.0045	0.0179	-0.0408	0.0273
Posterior Margin of Radii 5	5PMR1	13a	5	0.0-24.0	3.16†	6.24	-0.0536	0.0480	-0.0543	0.0004	0.1019
Number of Radii 5	5NR1	15	35	18.1-51.5	4.65	9.05	0.3334	-0.2297	-0.6029	0.4682	-0.0950
Disparity	Dp1	22	60	52.8-61.2	4.81	9.33	0.0066	0.0084	0.0081	-0.0007	0.0011
Scale 3 PC1	3PC1a	9	35	13.7-47.9	3.74	7.33	-0.0150	0.0022	0.0015	-0.0082	0.0089
Scale 3 PC2	3PC2a	7	50	33.7-57.9	5.04	9.80	0.0138	-0.0019	-0.0100	0.0119	-0.0038
	3PC2b	15	50	49.3-61.6	4.18	8.16	0.0003	-0.0063	0.0202	-0.0099	-0.0166
	3PC2c	4	60	43.6-62.0	4.36	8.51	-0.0166	0.0027	0.0068	-0.0117	0.0076
Scale 5 PC1	5PC1a	5	5	0.0-11.0	4.93	9.55	-0.0193	0.0095	0.0009	-0.0101	0.0186
Scale 5 PC2	5PC2a	7	45	33.7-57.9	6.11	11.71	0.0012	0.0010	-0.0065	0.0039	0.0037
	5PC2b	10a	10	2.6-21.5	3.40	6.69	-0.0181	0.0068	0.0015	-0.0098	0.0152
Scale 5 PC3	5PC3a	20	55	44.5-67.1	3.83	7.51	-0.0025	0.0010	0.0011	-0.0018	0.0017
